# Cooperation objective evaluation in aviation: validation and comparison of two novel approaches in simulated environment

**DOI:** 10.3389/fninf.2024.1409322

**Published:** 2024-09-17

**Authors:** Rossella Capotorto, Vincenzo Ronca, Nicolina Sciaraffa, Gianluca Borghini, Gianluca Di Flumeri, Lorenzo Mezzadri, Alessia Vozzi, Andrea Giorgi, Daniele Germano, Fabio Babiloni, Pietro Aricò

**Affiliations:** ^1^Department of Anatomical, Histological, Forensic and Orthopedic Sciences, Sapienza University of Rome, Rome, Italy; ^2^BrainSigns srl, Rome, Italy; ^3^Department of Computer, Control, and Management Engineering “Antonio Ruberti”, University of Rome “Sapienza”, Rome, Italy; ^4^Department of Molecular Medicine, Sapienza University of Rome, Rome, Italy; ^5^Urbe Aero Flight Academy, Rome, Italy; ^6^Department of Physiology and Pharmacology “Vittorio Erspamer”, Sapienza University of Rome, Rome, Italy; ^7^Department of Computer Science, Hangzhou Dianzi University, Hangzhou, China

**Keywords:** approach-withdrawal, cooperation, mutual information, circular correlation, electroencephalography, human factors, mental workload, neurophysiological

## Abstract

**Introduction:**

In operational environments, human interaction and cooperation between individuals are critical to efficiency and safety. These states are influenced by individuals' cognitive and emotional states. Human factor research aims to objectively quantify these states to prevent human error and maintain constant performances, particularly in high-risk settings such as aviation, where human error and performance account for a significant portion of accidents.

**Methods:**

Thus, this study aimed to evaluate and validate two novel methods for assessing the degree of cooperation among professional pilots engaged in real-flight simulation tasks. In addition, the study aimed to assess the ability of the proposed metrics to differentiate between the expertise levels of operating crews based on their levels of cooperation. Eight crews were involved in the experiments, consisting of four crews of Unexperienced pilots and four crews of Experienced pilots. An expert trainer, simulating air traffic management communication on one side and acting as a subject matter expert on the other, provided external evaluations of the pilots' mental states during the simulation. The two novel approaches introduced in this study were formulated based on circular correlation and mutual information techniques.

**Results and discussion:**

The findings demonstrated the possibility of quantifying cooperation levels among pilots during realistic flight simulations. In addition, cooperation time is found to be significantly higher (*p* < 0.05) among Experienced pilots compared to Unexperienced ones. Furthermore, these preliminary results exhibited significant correlations (*p* < 0.05) with subjective and behavioral measures collected every 30 s during the task, confirming their reliability.

## 1 Introduction

In an operational context, most human activities revolve around the interaction between two or more individuals engaged in group tasks. Consequently, efficiency and safety in the workplace depend not only on the individuals' performance but also on the collective workers' capability to cooperate with each other. In addition, the interaction between the individual and the surrounding working environment is significantly influenced by the individual's cognitive and emotional state. In the last decade, human factor (HF) research has focused on objectively quantifying these cognitive and emotional states as well as the way in which various states from different individuals interact, thereby influencing processes such as team communication, collaboration, and decision-making (Cooke and Gorman, 2009; Heaphy and Dutton, 2008). As a result, the capability to objectively evaluate the performance and cognitive resources of operators and their teams becomes critical to prevent fatal and serious human errors, especially in an operational environment (OE). It is important to highlight that OE refers to settings where people undertake particular tasks that demand technical and professional skills, wherein the level of risk must always be kept under control. In fact, according to National Transportation Board (NTSB) reports, in the last 20 years, ~85% of aviation accidents have been caused by human error (Risk and Management Handbook, 2022). Other statistics presented a slightly different percentage, ~69% (Waraich et al., 2013), but it remains significantly high and potentially preventable.

In this context, the HF related to cooperation could play a crucial role, especially within the field of aviation in which multiple operators are often asked to act as one by coordinating and finalizing each action to the same shared goal. Indeed, it has been demonstrated that in most types of interaction tasks, group-based systems perform better than individual-based and mixed systems, encouraging more cooperative behavior (Ladley et al., 2015). In addition, previous findings have elucidated an interesting phenomenon: Individuals who perceived heightened effort from their crew partner, particularly under high effort conditions, tend to invest more effort in the task and perform better. These findings are consistent with the idea that appreciating another person's effort increases one's own sense of commitment to a cooperative endeavor (Chennells, 2018). Thus, the capability to evaluate cooperation can lead to (i) improving performance, since better teams' performance can be induced by the awareness coming from evaluation of cooperation degree between members, (ii) improving safety, through the evaluation of cognitive and emotive mental state large scale mistakes can be avoided, while through the cooperation evaluation a constant degree of communication and collaboration can be maintained to avoid wrong management of emergency, and (iii) improving operators' wellbeing and satisfaction by increasing their confidence with respect to the environment, thereby contributing to improve performance too. These aspects are particularly valuable when applied in an OE as it is important that individuals' wellbeing and performance remain consistent to avoid human errors that could lead to serious consequences, including, in some cases, fatalities.

To date, especially in OE, scientific literature shows various investigated approaches for cooperation assessment. These approaches consist of methodologies based on subjective or behavioral measures (Ellis et al., 2023; Lapierre et al., 2023). Although subjective measurements are acknowledged to be useful in various contexts, it is important to be aware of the pertinent limits of such evaluations due to their inherent subjectivity and inability to capture the “unconscious” process underlying human behavior (Borghini et al., 2016; Dienes and Perner, 2004). In addition, to obtain these measurements from operators, it is necessary to interact with them, and this leads to difficulties to evaluate their state throughout particular activities. To address these limitations, in the last decade, technological developments have enabled the use of neurophysiological measurements also in OE (Borghini et al., 2012, 2020a; Vecchiato et al., 2014). As an example, measuring the brain and autonomic nervous system activities enables to obtain objective measures of specific mental states with low invasiveness and without negatively interfering with the operators (Arico et al., 2017). Moreover, since these kinds of measures are not subjective, the results could be generalized to assess the dynamic conditions of different subjects in the same operational environment.

Electroencephalography (EEG) and autonomic nervous system-based measures of workers' mental and emotional states have already been investigated during the recent decades to determine brain and autonomic cues of incoming risky psychophysical states (e.g., stress, drowsiness, inattention, and overload; Borghini et al., 2012, 2020a,b; Di Flumeri et al., 2022; Ronca et al., 2022; Vecchiato et al., 2014). Regarding cooperation assessment, the most recent scientific literature has demonstrated that specific indexes, such as functional connectivity and phase synchrony indexes, can reflect the state of cooperation between individuals (Réveillé et al., 2024). More specifically, the most proposed indexes to assess cooperation using EEG signals rely on functional connectivity indexes such as coherence and Granger causality, and synchrony indexes such as phase locking value (PLV), phase lag index (PLI), and circular correlation (Ccor; Réveillé et al., 2024; Sciaraffa et al., 2021). More importantly, these considered recent scientific contributions demonstrated how in the last decade cooperation evaluation through neurophysiological measures was conducted mostly in laboratory contexts and under hyperscanning conditions. The current limitations of functional connectivity indexes stem from the underlying hypotheses of these methods as they necessitate high-quality and high-density data, prolonged registration periods, and hyperscanning conditions to ensure accuracy (Bevilacqua et al., 2019; Liu et al., 2018; Richard et al., 2021; Toppi et al., 2016). In this regard, it has to be underlined that by hyperscanning condition, it is intended the one that foresees the simultaneous recording of brain electrical activity between individuals; hence, this experimental condition guarantees perfect time synchronization between signals. As presented earlier, other investigated methods to assess cooperation between individuals in the scientific literature are based on phase synchrony between signals (i.e., PLV, PLI, and Ccor). These methods aim to find similarities between EEG patterns over time that reflect the sharing of attention or psychological states necessary to coordinate actions aimed to achieve the same objective; phase synchrony also reflects a common response to the same environmental stimuli, similar actions aimed at the same goal, and similar engagement in the task (Burgess, 2013; Buzsáki and Wang, 2012; Stuldreher et al., 2022). Currently, these methods have exclusively been utilized within hyperscanning contexts (Wikström et al., 2022). However, the circular correlation (Ccor) method, due to its dependence on the circular covariance of differences between observed and expected phases, might demonstrate accuracy even in non-hyperscanning scenarios. Therefore, this is one of the approaches to be investigated by the present study.

A second methodology for the objective cooperation evaluation explored in the present study corresponds to one based on mutual information (MI). Such an approach relies on the information theory (IT; Cover and Thomas, 2006) according to the concept of Shannon entropy. The Shannon entropy measures the amount of information in a signal and provides an estimate of the level of uncertainty surrounding the signal-related event. In contrast to other indices based on IT, MI is capable of catching non-linear pattern similarity between two or more variables (Alonso et al., 2015); this property makes this methodology suitable for the study of EEG signals and their correlated features (i.e., mental states).

Considering the existing literature and taking advantage of modern technologies in terms of wearable and comfortable sensors, which can provide high-quality neurophysiological signals, the present study aimed to assess pilots' cooperation degree while performing flight simulations under realistic conditions through the use of neurophysiological measures. The cooperation assessment stands as a highly debated and challenging topic within scientific literature (Toppi et al., 2016). Currently, the lack of broadly validated methodologies, especially in real-world contexts, presents a substantial obstacle to the advancement of this research field. Particularly, for the application of these methods in OEs, it is important that the measuring system is non-invasive and compatible with environmental constraints, but at the same time it has to guarantee the high quality of the signals recorded. To address these needs, in this study, a wearable device for high-quality EEG data collection has been employed. Starting from this premise, the present study investigated the application and comparison of two novel techniques to assess cooperation between pilots during a real-flight simulation, contributing to state of the art by providing new methodologies for objectively evaluating cooperation in OE using mobile neurophysiological data collection systems. The potential innovation characterizing these methodologies corresponds to their robustness to experimental environments compatible with out-of-the-lab applications, which are notably violating the hypothesis of hyperscanning and lacking the high spatial resolution of traditional EEG measurements employed in controlled settings.

The present study is structured as follows: The selected methods for the cooperation neurophysiological characterization were technically described; subsequently, the experimental protocol, including all the collected data (i.e., neurophysiological, behavioral, and subjective measurements), was described; the experimental results and their conceptual implications were first presented and then discussed; finally, a conclusive section was presented to underline what are the main research outcomes of the presented study.

## 2 Materials and methods

As already mentioned in the Introduction section, the first proposed approach to evaluate cooperation was based on phase synchrony analysis of operators' EEG signals through the application of the circular correlation (Ccor) technique (Frassineti et al., 2021; Shahsavari Baboukani et al., 2019).

The second approach was based on empirical evidence suggesting that cooperation can be assessed as the output of a multivariate system derived from the cognitive and emotional states of users (Sciaraffa et al., 2021). Such a multivariate system was therefore computed through the MI. In this regard, the cognitive and emotional states were associated with the mental workload (MW) and approach-withdrawal (AW), respectively. More specifically, EEG-derived indexes for MW and AW have been selected for each individual engaged in the cooperative task since the MW reflects the level of cognitive effort, while the AW indicates the emotional tendency to approach or withdraw from a stimulus.

To technically validate both the proposed approaches, simulated crews (FAKE crews) were constructed, randomizing pilots within the crews and experimental phases so that their input in the method would be considered white noise in terms of cooperation. These simulated crews served to highlight the sensibility and reliability of the proposed indexes to cooperative behavior, as by construction, they cannot reflect cooperative behaviors. Therefore, it was expected that these indexes would consistently demonstrate a fixed response pattern for FAKE crews, whereas for REAL crews they must be sensitive to the cooperation phase. The choice of Ccor and MI-based approaches among all the most investigated and validated methodologies within the state of the art is motivated by their ability to capture both linear and non-linear relationships between time series, providing a more comprehensive view of information exchange. It is important to note that both the proposed methods investigate the same phenomenon from complementary perspectives. To standardize the terminology in the present study, the cooperation index assessed through mutual information will be referred to as the MI-Cooperation Index (MICI), while the one assessed through circular correlation will be termed the Ccor-Cooperation Index (CCI).

### 2.1 Cooperation assessment

As previously mentioned, the cooperation index [C(t)] over time has been computed through two approaches that will be described along the respective following subchapters.

#### 2.1.1 Ccor-Cooperation Index assessment

CCI method is based on the similarity between the instantaneous phases of two time series which are directly the EEG signals from each pilot involved in the task. Therefore, the CCI was computed according to the following formula (Berens, 2009; Shahsavari Baboukani et al., 2019).


(1) $$Ccor_{\vartheta ,\varphi }\;=\;\frac{\sum_{k=0}^{N-1}\sin(\vartheta_{k}-\;\bar{\vartheta })\sin(\varphi_{k}-\;\bar{\varphi })}{\sqrt{\sum_{k=0}^{N-1}\sin^{2}(\vartheta_{k}-\;\bar{\vartheta })\sin^{2}(\varphi_{k}-\;\bar{\varphi })}}$$

- ϑ_*k*_ and φ_*k*_: instantaneaous phases evaluated at k-th sample;
- $\bar{\vartheta }$ and $\bar{\varphi }$: circular means of ϑ_*k*_ and φ_*k*_ computed using 2.

(2) $$\bar{\vartheta }=\arg\left(\sum_{n=0}^{N-1}e^{j\;\bar{\vartheta }[n]}\right)$$

Phases have been considered in radians.

CCI has been computed first on the overall preprocessed signal and second on each individual frequency band as reported in Table 1. For each individual, individual alpha frequency (IAF) was computed on the eyes closed EEG signal (Klimesch, 1999).

**Table 1:** Frequency band definition.

Theta	[(IAF – 6): (IAF – 2)] Hz
Alpha	[(IAF – 2): (IAF + 2)] Hz
Beta	[(IAF + 2): (IAF + 16)] Hz

The percentage of cooperation time has been computed by determining how long the cooperation neurophysiological index was above the cooperation threshold. The cooperation threshold has been established as the CCI median evaluated along the calibration tasks (i.e., low and high difficulty).

#### 2.1.2 MI-Cooperation Index assessment

Considering the two discrete variables of MW and AW, the theoretical formulation of MICI is described by the following formulas (Cover and Thomas, 2006; Kraskov et al., 2004):


(3) $$I\left(X;Y\right)=\;\sum_{i,j}p\left(i,j\right)\log\frac{p\left(i,j\right)}{p_{x}\left(i\right)p_{y}(j)}$$

(4) $$I\left(X;Y\right)=\;\iint p\left(x,y\right)\log\frac{p\left(x,y\right)}{p_{x}\left(x\right)p_{y}\left(y\right)}dxdy$$

where p(i,j) is the joint probability of the two discrete time series, *p*_*x*_(*i*) and *p*_*y*_(*j*) are the marginal probabilities of each time series, and i,j are bins of the 2D discrete space in which the two series are defined.

The reported discrete formula (3) is already an approximation of the continuous MI evaluated on a continuous space (4). To technically implement the aforementioned formulation, Kraskov et al. (2004) proposed an estimator of MI based on entropy (H) concept:


(5) I(X;Y)= H(X)+H(Y)−H(X,Y)

Thus, MICI(t) was assessed as the mutual information between four time series (i.e., MW index and AW index assessed on both individuals), using the Kraskov estimator (Kraskov et al., 2004). This is a KNN-based estimator that aims to estimate the Shannon entropy of every signal from the average distance of the k-th nearest neighbor averaged over all other points of the signal. Since the computation of mental workload and approach-withdrawal indices involves the evaluation of power across distinct EEG signal bands, the analysis of the MICI index's contribution was constrained to the entire preprocessed signal's band, precluding examination within specific frequency bands like done with CCI.

The percentage of cooperation time has been computed as according to the procedure described in the previous paragraph.

### 2.2 Experimental protocol design and participants

Sixteen (16) participants have been selected and organized in eight flight crews, four of which were composed of Unexperienced pilots (UNEXPs, i.e., pilots that had just got the integrated ATPL—Airline Transport Pilot License to become commercial pilots) and four by Experienced ones (EXPs, i.e., pilots working for a commercial airline for at least 10 years). Every crew was flanked by an expert trainer with the role of supervising and providing information about the pilots' mental states while performing the experimental tasks. Furthermore, to mimic a more realistic situation, the expert trainer was designated to act as an air traffic controller to simulate the communications with the crews. The flight simulations were performed on the Mechtronix Ascent XJ Trainer Boeing 737–800 simulator, located at the Urbe Aero training center in Rome.

**Figure 1:**
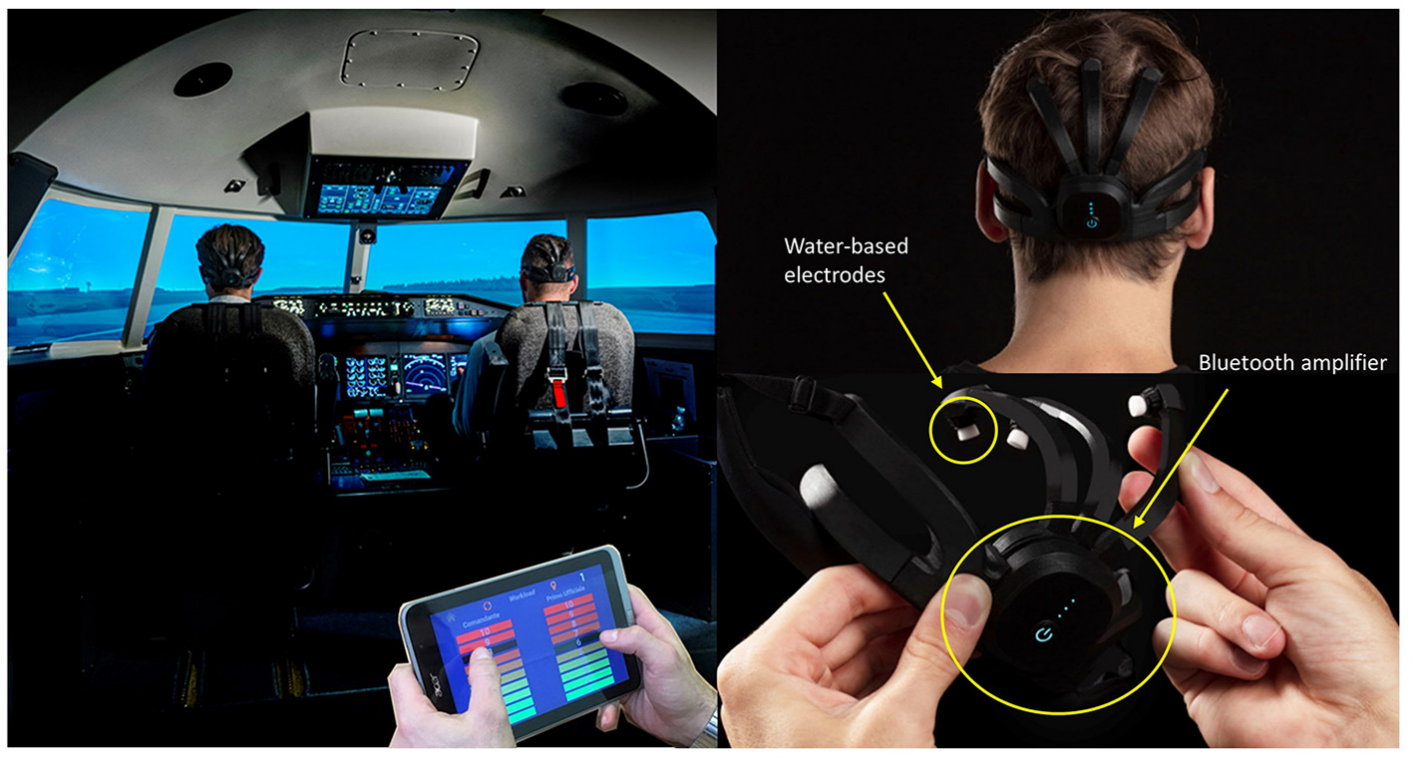
On the right is shown the experimental setting in the Mechtronix simulator and on the left the Mindtooth Touch EEG headset (BrainSigns srl, Rome, Italy). The system has been designed to be wearable, comfortable for a long use, easy to wear, and fully compatible with aviation tools (e.g., headphones and glasses).

The experimental protocol was organized into two major sections described in Table 2. In the first phase, defined as the “calibration scenario,” each pilot performed the experimental flight simulation alone. In the second phase, both pilots shared the same cockpit to perform a mission designed to induce cooperative behavior between them. The pilots' EEG signals were collected throughout the two mentioned experimental phases. The first scenario, which was completed by each pilot alone, was designed to derive a sort of baseline in an uncooperative environment. This phase was necessary to determine the effective cooperation threshold for each pilot crew. In addition, two preliminary 60-s EEG data collections were performed, while the participants were relaxed with the eyes closed, to evaluate the individual alpha frequency (IAF; Klimesch, 1999) for each individual, and while the participants were laying still relaxed, with the eyes open for calibrating the eyeblink correction algorithm (Di Flumeri et al., 2016). At the end of each flight mission, the trainer who supervised all the activities provided subjective behavioral assessments every 30 s of the pilots' cooperation effectiveness through a rating scale on a tablet (Figure 1). According to the trainer's behavioral assessment, a performance index representative of the outcome of the mission and the mental condition of each crew was assessed as described in Section 2.4.

**Table 2:** Experimental Protocol's scenario description.

**Phases**	**Description**
Calibration scenario	The pilots were asked to conduct an approach and landing with low visibility during two adverse simulated weather conditions, so at two different difficulty levels—low difficulty (Low diff) and high difficulty (High diff) phases—*each pilot has conducted this phase individually*.
Cooperation scenario	During this session, a failure on the landing gears has been injected during the approach phase. This section has been designed to induce cooperative behaviors—*This phase was conducted by two pilots at a time, working together as a crew*.

### 2.3 Neurophysiological data recording and processing

The Mindtooth system (Brain Products GmbH, Gilching, Germany, and BrainSigns srl, Rome, Italy) has been employed for EEG signal acquisition. This system was developed within the homonymous project (H2020-EIC-FTI-GA950998), and it is able to record high-quality EEG data while maintaining high comfort for the operator (Sciaraffa et al., 2022). The EEG data were collected with a sample rate of 125 Hz, on eight recording channels (AFz, AF3, AF4, AF7, AF8, PZ, P3, and P4), referenced to the right mastoid, and grounded on the left one. Electrodes' impedance has been maintained below 50 kOhm (Sciaraffa et al., 2022). All measurements were subjected to a 50 Hz notch filter to remove main line power interference. AF7 and AF8 channels were removed from the analysis due to a too high percentage of movement artifacts coupled with the EEG signal, while the other channels were preserved with an average artifact percentage below 8% as shown in Table 3.

**Table 3:** Mean artifact percentage on each preserved channel.

**Channel**	**Mean artifact %**
AF3	6.89
AF4	7.93
AFz	7.93
P3	6.40
P4	5.33
Pz	5.66

The EEG recordings were also band-pass filtered [low-pass filter cutoff frequency: 30 Hz, high-pass filter cutoff frequency: 2 Hz] with a 5-th order Butterworth filter. The Reblinca method was used to identify the blink artifacts and the ocular component, which were corrected using a multi-channel Wiener filter (MWF; Di Flumeri et al., 2016). EEG signals were segmented into epochs of 1 s, and on every epoch, only the threshold criterium was applied to mark artifactual epochs through the EEGLAB toolbox (Delorme and Makeig, 2004). In particular, the EEG epochs were identified as “artifact” if the EEG amplitude was >±80 (μV). Once the artifact-free EEG signal was computed, global field power (GFP) was estimated for each frequency band as a preliminary step to compute the evaluation of cooperation-related neurophysiological metrics (i.e., MW and AW). Frequency bands have been defined in Table 3.

The GFP-derived (Skrandies, 1990; Vecchiato et al., 2014) features computed among the aforementioned frequency bands and EEG channels have been used to assess mental workload index [W(t)] and approach-withdrawal index [AW(t)] over time:


(6) $$W\left(t\right)=\;\frac{GFP_{\vartheta }\left(AF3,AFz,AF4\right)}{GFP_{\alpha }\left(P3,Pz,P4\right)}$$

(7) $$AW\left(t\right)=\;GFP_{\alpha }\left(AF4\right)\;-\;GFP_{\alpha }\left(AF3\right)$$

where *GFP*_*x*_ represents the GFP evaluated on the individual x-band.

**Figure 2:**
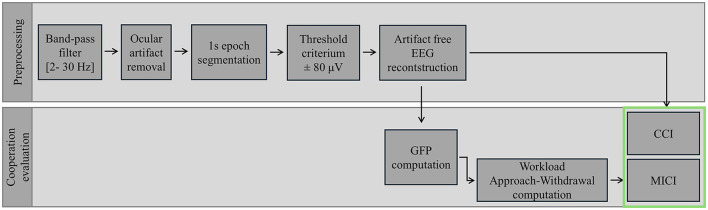
Overview of the preprocessing steps and analytical methods applied to EEG signal analysis, including noise reduction, artifact removal, feature extraction, and cooperation index evaluation. Cooperation indexes are highlighted by a green framework.

The overall preprocessing and cooperation evaluation process is graphically presented in Figure 2.

### 2.4 Behavioral data

As previously anticipated, during each flight mission, an instructor, acting as subject matter expert (SME), supervised all the activities and provided a subjective assessment of cooperation degree between pilots by a rating scale on a tablet (Figure 1) every 30 s.

Crew's total behavioral cooperation assessment was computed as the mean between the behavioral cooperation assessment of the pilots composing the crew.

### 2.5 Statistical analysis

The Shapiro–Wilk test was used to assess the normality of the distribution related to each of the considered parameters evaluated through the pilots' dyads. If normality was confirmed, Student's unpaired *t*-test would have been performed to pairwise comparing Experienced (i.e., EXPs) and Unexperienced (i.e., UNEXPs) groups. Otherwise, the same differences have been tested through the Mann–Whitney test.

ANOVA test has been applied in different phase comparisons after verification of the ANOVA assumptions.

In addition, to validate the proposed indexes, the relationships between each neurophysiological cooperation index (i.e., CCI and MICI) and crews' behavioral cooperation assessment were examined using Pearson's correlation analyses. Furthermore, an additional validation method involved comparing the results obtained from REAL and FAKE crews, assuming that the methods applied to the FAKE crews would return constant results. This last analysis was conducted using both ANOVA and Student's unpaired *t*-test to compare between phases and between REAL and FAKE in each phase.

## 3 Results

The result paragraph has been organized in two different subsections to neatly distinguish the results from different methodologies. The results from MI and Ccor methodologies to assess cooperation (i.e., the CCI and MICI) will be presented, respectively, on the first and second subchapters.

### 3.1 Mutual information approach: MICI results

**Figure 3:**
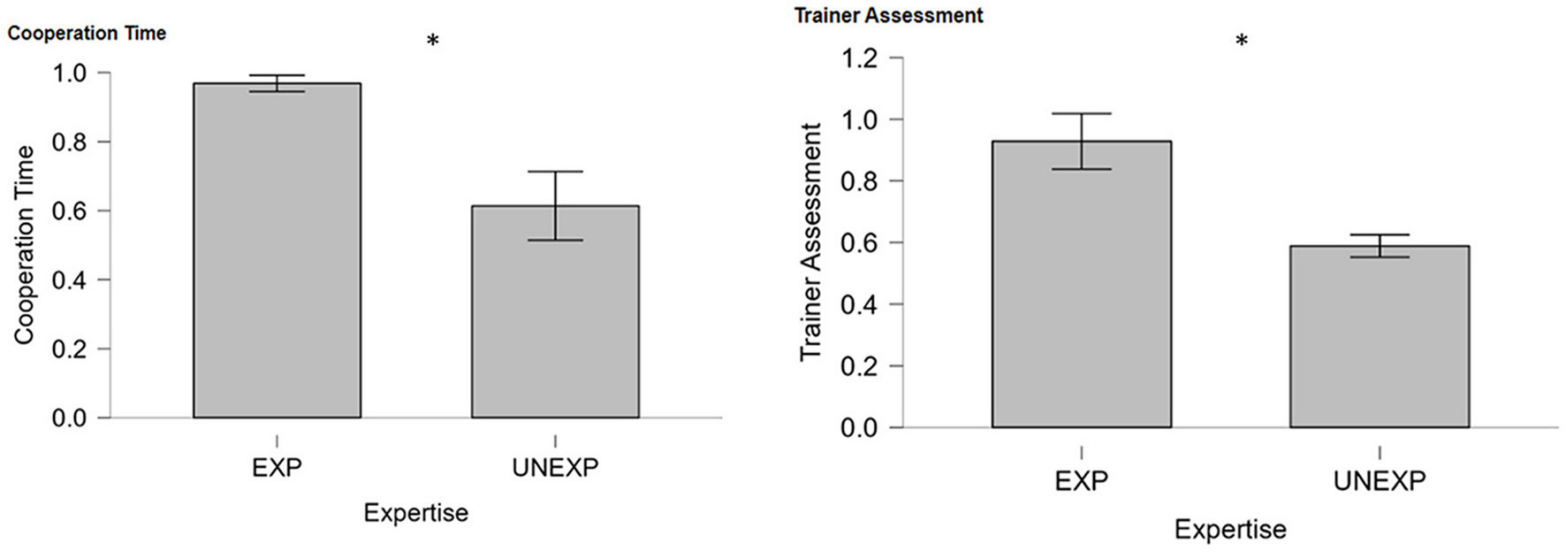
Percentage of cooperation time evaluated through MICI and trainer performance assessment between EXP and UNEXP pilots. *Indicates the statistical differences between experimental groups (both *p* < 0.05).

Two statistical unpaired Student's *t*-tests, regarding the difference in cooperation time and performance between EXPs crews and UNEXPs ones, have been performed as shown in Figure 3. The first analysis (left in Figure 3) aimed to compare average cooperation time in the two experimental groups, which was found to be statistically higher in the EXP group (*p* = 0.017). The second analysis presented on the right side in Figure 3 compares instead the cooperation assessment provided by the trainer in the same experimental groups, resulting once more to be statistically higher in the EXP group (*p* = 0.015). As shown, both neurophysiological and behavioral assessments reveal the same trend in the two experimental groups suggesting that the measurements truly observe directly related phenomena such as evaluated teamwork and tracked performances.

**Figure 4:**
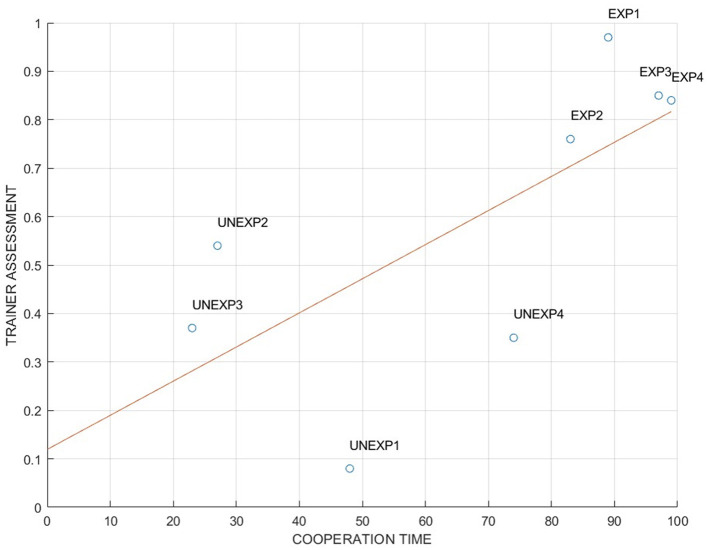
Pearson's correlation between cooperation time and trainer performance assessment (*R* = 0.78, *p* < 0.05).

The Pearson correlation analysis in Figure 4 conducted between the two cooperation measures (i.e., MICI and the SME assessment) has shown a high positive and significant correlation, confirming that the MICI index follows the judgment of the SME in evaluating cooperation (*R* = 0.78, *p* = 0.032). This result particularly suggests that the two measures describe the same phenomenon (i.e., neurophysiological and behavioral cooperation) and serve to validate MICI index as a representative cooperation index.

**Figure 5:**
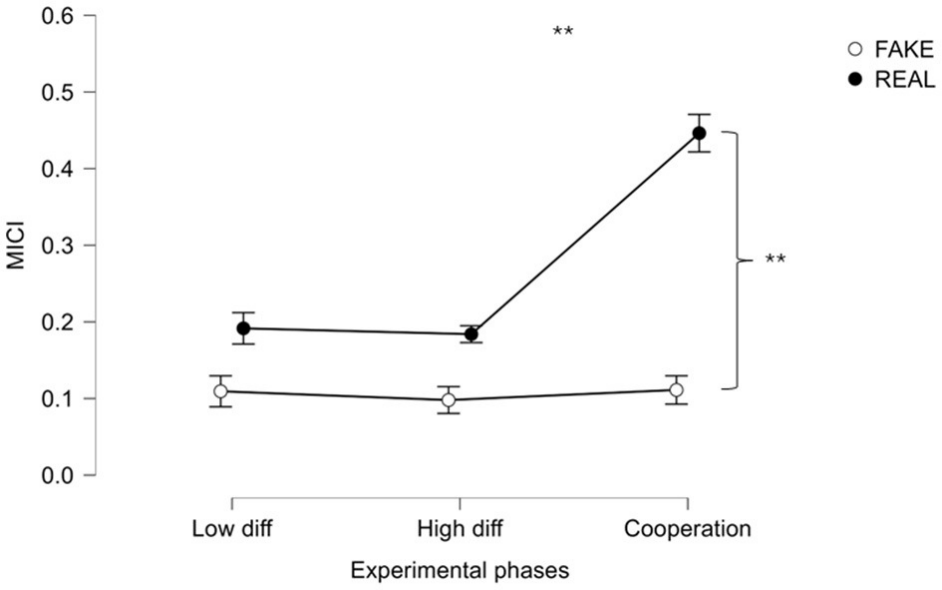
ANOVA comparison of MICI through different experimental phases in real and simulated crews. **Indicates the statistical difference in the behavior during the experimental phases between real and simulated crews (both *p* < 0.001).

Figure 5 represents the REAL vs. FAKE crews ANOVA analysis revealing statistically significant (*p* < 0.001, *F* = 28.697, ω^2^ = 0.491) differences in behavior along phases. In addition, the *t*-test between REAL and FAKE on cooperation phase resulted in a significant difference (*p* < 0.001).

### 3.2 Circular correlation approach: CCI results

All the presented results regarding CCI are assessed on the overall signal band (i.e., 2–30 Hz). Individual power band analysis did not result in significant results.

**Figure 6:**
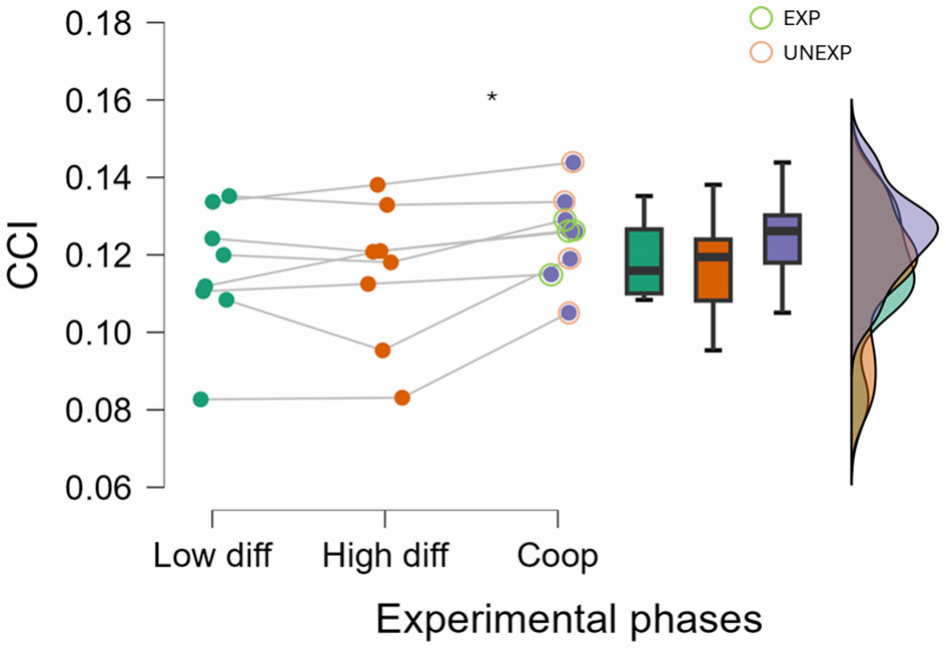
Raincloud of CCI through different experimental phases on the frontal electrodes. *Indicates the statistical difference between the cooperation condition and the solo ones (i.e., Low and High diff; *p* < 0.010).

Figure 6 shows the Friedman statistical comparisons between cooperative experimental conditions and non-cooperative ones (i.e., Low and High diff). This statistical analysis demonstrated that the CCI was significantly higher when evaluated along the cooperative experimental tasks compared with the non-cooperative ones (*p* = 0.005, *F* = 7.833, ω^2^ = 0.061) over the frontal area.

**Figure 7:**
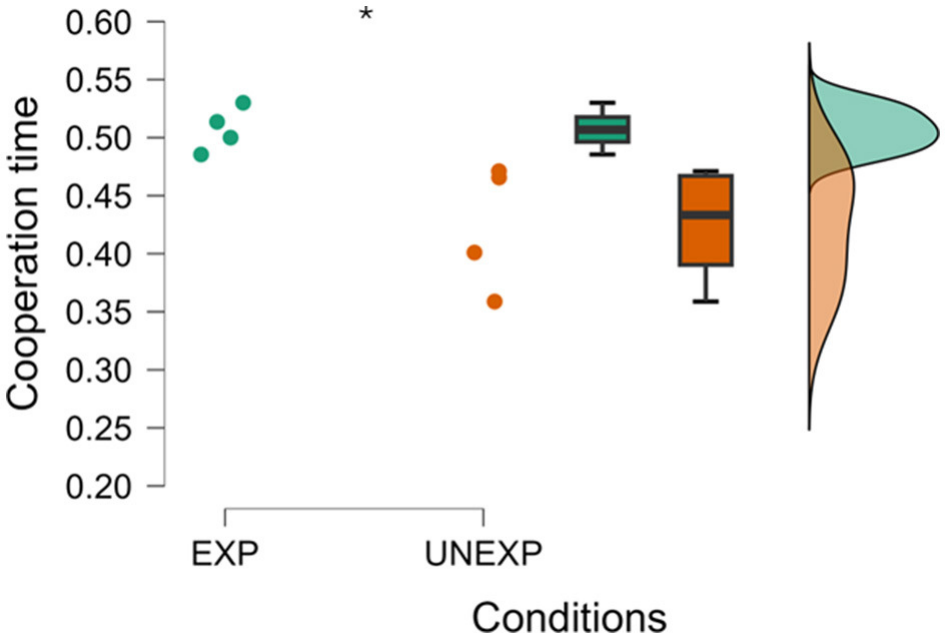
Raincloud plot of percentage of cooperation time between the experimental groups on frontal electrodes. *Indicates the statistical difference between the EXP and UNEXP condition (*p* = 0.029).

By considering the second dimension of comparison, that is, distinguishing between pilot categories EXPs and UNEXPs based on their cooperation levels, the statistical analysis revealed a significant difference in the percentage of cooperation time between the two groups, with EXP crews demonstrating notably higher cooperation time on frontal electrodes compared to the UNEXP group (*p* = 0.029, *W* = 16.000). This distinction is represented in Figure 7, illustrating the percentage of cooperation time assessed on frontal electrodes, and the Mann–Whitney test has been used for this analysis. The same analysis conducted on parietal electrodes did not report statistically significant evidence.

**Figure 8:**
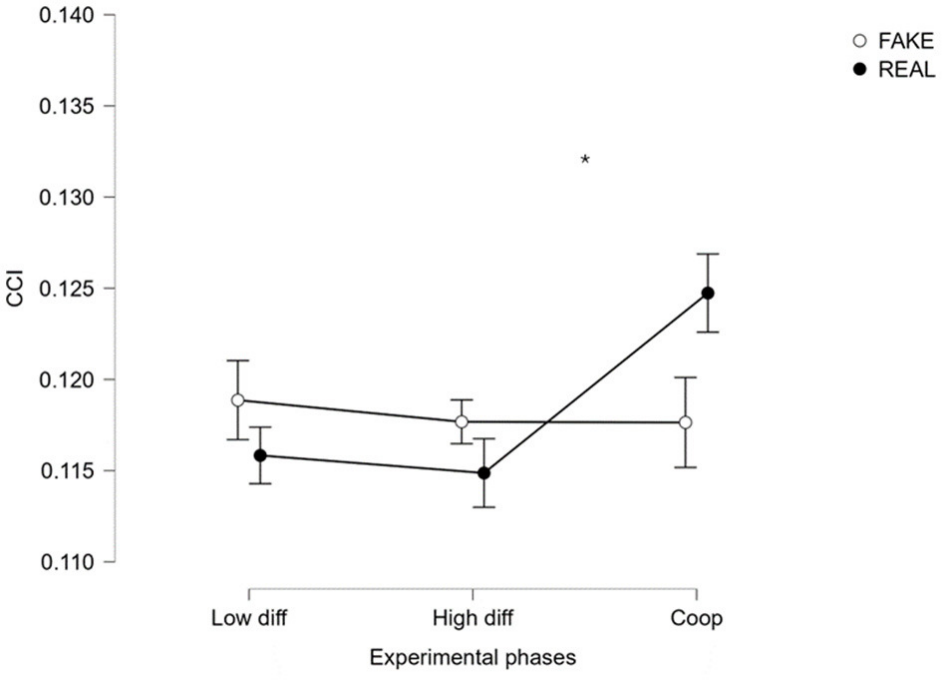
ANOVA comparison of CCI through different experimental phases in real and simulated crews. *Indicates the statistical difference in the behavior during the experimental phases between real and simulated crews (*p* = 0.022).

Furthermore, to validate such Cooperation index computed through CCor, an ANOVA comparison between REAL crews and FAKE crews has been conducted (Figure 8), demonstrating statistically significant (*p* = 0.022, *F* = 4.340, ω^2^ = 0.020) differences in behavior between real and simulated crews during the experimental phases. Unfortunately, the Pearson correlation analysis conducted between CCI and SME assessment did not return any statistical correlation (*R* = 0.3854, *p* = 0.3458).

As stated earlier, all the presented statistical analyses were also conducted according to each frequency band of the EEG signal described in Table 1 aiming to identify any differences in the behavior of the CCI across bands. The results underlined any statistically significant outcomes.

## 4 Discussion

In this study, the main aim was to investigate the effectiveness of two indices based on neurophysiological measures in quantifying the degree of cooperation of crews of pilots during flight operations. Both methodologies investigate the same phenomenon, but they present very different properties. The MI approach provides a psychological interpretation of the pilots' mental state during the task, yet it lacks temporal resolution as it relies on a double-derived measure from the EEG signal. In contrast, the Ccor approach offers optimal temporal resolution as it is directly derived from the EEG signal, but its results reflect neurophysiological cortical activation, whereby it does not provide a psychological interpretation.

In particular, the result presented in Figure 3 showed that the EXP level of cooperation evaluated through MICI was higher than in UNEXP pilots in both neurophysiological and SME's evaluation. This result was expected since experienced pilots have received intense training in their flight company (the same for each couple of EXP pilots), oriented to reinforce cooperative behaviors between captain and first officer. Consequently, even if two pilots who do not know each other are required to collaborate, the cooperation during the flight operation is still high. Concerning the validation of the MICI index, it is important to note that the correlation analysis presented in Figure 4 consistently revealed a significant and high correlation value between neurophysiological and behavioral cooperation evaluations. In addition, the comparison between REAL and FAKE crews presented in Figures 5, 8 shows that the two cooperation indexes were sensitive to cooperative behavior (i.e., during the scenario in which the two pilots had to cooperate). This aspect has been confirmed by a significant decrease of the indexes, if calculated over the calibration data (each pilot worked alone). The consistent response to white noise input, observed in FAKE crews' analysis, further underscores the sensitivity of these indexes in capturing real cooperation, thus strengthening their validation. These results align with our initial hypotheses and provide further support for the validation of the indexes.

Concerning the circular correlation-based approach, the results presented in Figure 6 showed that the method can be used for the cooperation evaluation between two subjects as it is able to capture, by considering the EEG channels located among the frontal area, the difference between cooperative (i.e., cooperation) and non-cooperative conditions (i.e., Low and High diff). The results were also statistically significant (*p* = 0.005) despite the limited quantity of experimental samples (i.e., 8 dyads). These results computed by considering the EEG frontal channels were also supported by the analyses shown in Figure 7. Percentual time of cooperation evaluated on frontal electrodes resulted to be statistically higher (*p* = 0.029) in the EXP group respect to UNEXPs one, as already assumed a priori and confirmed by trainer assessment (Figure 3). On the contrary, over the EEG parietal channels, there were no statistically significant results. This did not support the hypothesis of a major evaluated cooperation degree within the cooperative experimental phase (i.e., cooperation) with respect to the non-cooperative ones (i.e., Low and High diff). This result appears to be robustly coherent with the cooperation time percentage assessed over the EEG parietal channels, which did not demonstrate a statistically significant difference, further highlighting the lack of support for the hypothesized increase in cooperation during the experimental phase. These findings suggest that, at the best of our knowledge based on these preliminary results, the sensitive region in terms of cooperation evaluated through CCI is the frontal one.

The lack of validation for CCI via correlation with trainers' behavioral assessment, which revealed a positive correlation (*R* = 0.38) even if not statistically significant, may be due to the conceptual definition of the CCI. In fact, the CCI was computed according to the EEG signal phase similarities between the two operators cooperating, while the MICI was defined according to the cognitive and emotional state synchronies between the operators cooperating. Therefore, it can be hypothesized that the SME subjective evaluation of the cooperation grade, which obviously relied on the SME's cognitive and emotional perceptions, was more coherent with the MICI than the CCI. Moreover, it has to be considered that the two proposed methods are characterized by different temporal resolutions and that the MICI, which was computed according to the MI involving cognitive and emotional states (i.e., the MW and AW) calculated in the frequency domain, was the one among the two more comparable with the SME subjective evaluations in terms of temporal resolution.

A spectral analysis was conducted within specific frequency bands to examine the hypothesis to explore whether certain bands of the EEG signal contribute significantly to cooperation assessment (only for CCI as described in Sections 2.1.1 and 2.1.2). Unfortunately, this last analysis reported no statistical significance, and this may have been caused by the small effect size of the phenomenon merged with the weak statistical power of the conducted analysis due to the small amount of analyzed dyads.

It is crucial to acknowledge the limitations encountered in this last analysis. In the case of MICI, a key limitation arises from the methodology itself, restricting the analysis of cooperation exclusively to the overall signal. This constraint is inherent in the derivation of MICI from the mental workload and the approach-withdrawal measures, which, by definition, concentrate on specific frequency bands. Consequently, the application of MICI cannot extend to a custom frequency range but must adhere to the requirements of the provided inputs, highlighting a methodological limitation.

On the other hand, while the methodology of CCI does not inherently impose limitations, the present results do not conclusively demonstrate its capability to capture distinct cooperation behaviors corresponding to the different frequency bands characterizing the EEG. To resolve this uncertainty, future studies will exploit a more comprehensive approach, employing a larger sample size to assess the CCI's ability to investigate cooperation across various frequency bands.

Another interesting consideration can be done regarding the potential application of machine learning-based techniques in this research field. In fact, since the proposed MICI approach involves the research of linear and non-linear combination of various neurophysiological features, the application of machine learning could be promising, although it has not yet been extensively explored by the scientific community. Unfortunately, in the context of the presented research, it was not possible to investigate a machine learning approach due to the lack of data, since this kind of algorithm requires large amount of data for training and testing. Furthermore, considering the realistic environment and the particularly specialized experimental sample, such as real-flight pilots, it is even more challenging to collect data on a scale sufficient to enable a machine learning approach. Indeed, this aspect constitutes one of the most promising potential directions for future research in the context of the cooperation neurophysiological modeling.

## 5 Conclusion

The presented neurophysiological-based indexes (i.e., the MICI and CCI) were designed to overcome the limitations characterizing the subjective measurements highly investigated in scientific literature as cooperative dynamics indicators, even in OE. The cooperation neurophysiological assessment could mitigate the subjective' inability and bias for capturing the unconscious mechanisms of human behavior (Borghini et al., 2017; Dienes and Perner, 2004).

Based on these results, it is evident that objectively quantifying cooperation among pilots involved in real tasks through neurophysiological-based methods is achievable. The higher level of cooperation observed over the EEG channels located within the frontal area among experienced pilots supports the initial hypothesis, which suggested that Experienced (i.e., EXP) who underwent a cooperation training course would exhibit enhanced cooperative skills. On the contrary, the results obtained by applying the proposed CCIs over the EEG parietal channels and the frequency analysis encourage a more complete investigation by considering a larger sample size to support the statistical analysis. Thus, this result obtained from CCI suggests that the frontal area is the most involved area in cooperative processes, at least in this specific environment. This hypothesis is confirmed by the fact that both features used to evaluate MICI (i.e., MW and AW) are evaluated as combination of GFP over bands specifically on the frontal electrodes, confirming what frontal CCI analysis found.

Substantially, these results demonstrated how the MICI and CCI endow promising methods for an objective evaluation of users' cooperation degree while dealing with realistic tasks since their capability for statistically discriminating between cooperative and non-cooperative behaviors.

The presented study provides advancement with respect to the cooperation estimation state of the art. In fact, the presented approach demonstrated to be reliable in evaluating brain dynamics within realistic scenarios and sensible to different cooperation grades (i.e., experienced and non-experienced pilots engaged in flight simulation tasks), regardless of hyperscanning constraints. It is important to note that, among the scientific literature, the utilization of a phase synchrony-based index such as Ccor to assess brain synchronization has been addressed only in a limited way, and it has not been employed to evaluate cooperation in real environments. This field represents the focus of the current research, aspiring to make a substantial contribution. However, it is important to acknowledge certain limitations of the proposed indexes such as their inability to underline different frequency band contributions to the cooperation estimation.

In conclusion, these findings highlight the potential of neurophysiological measures as valuable indicators of cooperation in operational environments. The ability to objectively quantify these aspects opens opportunities for targeted interventions, training programs, and improvements in team dynamics. By integrating both objective and subjective measures, a more comprehensive understanding of teamwork can be achieved, facilitating the identification of factors that contribute to effective collaboration and ultimately enhancing performance and outcomes in high stake tasks. This will contribute to improving the wellbeing, safety, and performance of working teams, allowing both public and private industries to achieve greater efficiency, which saves time, money, and limits environmental impact in specific cases. For example, in pilots' training, the capability to monitor the emotional and mental state, as well as the cooperation degree during training, would allow for the creation of individual customized training sessions, by consequently reducing the team's total flight time required for the training completion. Moreover, within working industrial environments, such as the manufacturing industry, the cooperation and continuous monitoring among team members would enable optimized and more efficient dynamic management of work schedules, ensuring that tasks' execution would be concentrated exclusively within high-performance workflows. This approach would certainly reduce downtime and inefficiencies within industrial operational processes. Such promising results pave the way toward employing wearable EEG systems, such as the one used in this study, for monitoring operators in their operational environment. Taking specifically into account pilots, the capability to objectively quantifying cooperation can lead to improvement in training program, security, and efficiency.

## Data Availability

The data presented in this article are not readily available as since the data were collected as part of a European project, and given that they involve sensitive neurophysiological information, their availability is contingent upon approval from the consortium as well as the explicit consent of the participants involved. The authors are open to considering data sharing requests under reasonable request.
